# Anticipatory Stress Increases Deontological Inclinations: The Mediating Role of Emotional Valence

**DOI:** 10.3390/bs12120476

**Published:** 2022-11-24

**Authors:** Zhongquan Li, Liuping Gao, Lisong Zhang

**Affiliations:** 1 Department of Psychology, School of Social and Behavioral Sciences, Nanjing University, Nanjing 210023, China; 2 Jiangsu Provincial Education Examination Authority, Nanjing 210036, China; 3 School of Sociology and Population, Nanjing University of Posts and Telecommunications, Nanjing 210049, China

**Keywords:** acute stress, moral judgment, utilitarian inclinations, deontological inclinations, emotional valence

## Abstract

Previous studies have explored the differences in moral judgments under normal situations and acute stress using the Trier Social Stress Test (TSST). The present study examined whether anticipatory stress (i.e., induced by an anticipated speech) could elicit similar effects and further explored the mediation of emotional responses between acute stress and moral judgments with a process-dissociation approach. Fifty-three undergraduate students (20 males and 33 females) were randomly assigned to the stress and control groups. In the first stage, they were instructed to prepare a public speech (the stress group) or just recall events during the previous vacation (the control group). In the second stage, they reported emotional valence and arousal for each moral dilemma in a set of 12 moral dilemmas, followed by judgments on moral acceptability of the agent’s action. The manipulation check confirmed that anticipatory stress was reliably induced, as indicated in both self-reported and physiological data. The traditional dilemma analysis revealed that participants in the stress group would make fewer utilitarian judgments than those in the control group. The process dissociation (PD) analyses further revealed that the stress group exhibited higher deontological inclinations than the control group, but no significant differences in utilitarian inclinations. Emotional valence played a mediating role in the association between stress and deontological inclinations. To sum up, our study extended the investigation of the relationship between acute stress and moral judgment to anticipatory stress, clarified its distinct impact on deontological and utilitarian inclinations, and revealed the mediating effect of emotional valence.

## 1. Introduction

Stress is relevant to our lives, and we often have to make many important judgments and decisions in stressful scenarios, for example, judgments and decisions in the moral domain [1,2]. There has been a large body of research demonstrating that stress has significant effects on our cognitive and emotional processes, and affects decision-making in many areas [2]. However, empirical studies on the relationship between stress and moral decision-making have started late [3], and this topic has received increasing attention from scholars since then [1,4,5,6].

Most previous researchers have used the Trier Social Stress Test (TSST) paradigm to induce acute stress and then investigated the differences in judgments on abstract moral dilemmas between the stress and control groups. Youssef et al. [6] compared the responses of the stress group and the control group to non-moral, impersonal moral, and personal moral dilemmas. The results indicated that the stress group made less utilitarian responses to personal moral dilemmas. Li et al. [7] further addressed the interpretational ambiguities of traditional analysis with the CNI model approach. They found that the stress group made more deontological judgments than the control group in traditional dilemma analysis. The Consequences, Norms, and Generalized Inaction (CNI) model analysis revealed that the stress group indicated a stronger sensitivity to moral norms and a higher general preference for inaction than the control group. Some researchers have also attempted to use other types of stress-inducing paradigms to explore the effects of stress on moral judgment, such as time pressure and anticipatory stress. Suter et al. [8] used a temporal stress elicitation paradigm to manipulate the stress levels. The participants in the stress group were required to respond quickly within 8 s, while the control group was given 3 min to think about their answers. The stress group made more deontological judgments on self-involved moral dilemmas. Cummins et al. [9] similarly found that limiting decision time reduced the proportion of utilitarian judgments. However, [10] explored the effects of intuitive thinking on moral judgments using both time pressure and cognitive load in a large sample of 1413 participants, but they did not find an effect of time pressure and cognitive load on moral judgments. Starcke et al. [5] employed an anticipatory stress elicitation paradigm with 50 college students with heart rate as a physiological indicator and had participants respond to moral dilemmas. The results showed that participants in the stress group made fewer utilitarian judgments than those in the control group and that the “non-self-involving” moral dilemma was more likely than the “self-involving” moral dilemma. In addition, an increase in heart rate was significantly and negatively associated with utilitarian judgment. More recently, some researchers have attempted to address the issue of how acute stress exposure affects everyday moral decision-making and the roles of gender, personality, social closeness, and timing [11,12]. They found that the stress group made more altruistic decisions than the control group. Moreover, participants made more altruistic decisions toward socially close targets (e.g., mothers) than toward socially distant targets (e.g., strangers).

In these studies, a traditional data analysis approach to moral dilemmas has often been used (with the exception of [7]). This approach has some shortcomings [13]. Participants are often presented with a series of moral dilemmas, and then they are asked to make judgments about the acceptability of the agent’s moral action. Deontological or utilitarian judgments are scored by calculating the proportion of responses in which participants consider the action to be unacceptable or acceptable. The higher the proportion of utilitarian actions unacceptable, the more deontological judgments are favored [4,5,14,15]. This approach treats deontological and utilitarian judgments as the opposite, which violates the assumption suggested by the Dual-Process Theory of Moral Judgment (DPTMJ) that deontological and utilitarian principles stem from two independent processes [16]. Moreover, this approach cannot distinguish whether individuals make deontological (utilitarian) judgments because deontological (utilitarian) inclinations are stronger or because utilitarian (deontological) inclinations are weaker. To overcome these problems, P Conway and B Gawronski [17] teased apart the distinct contributions of deontological and utilitarian inclinations by applying the process dissociation approach (PD) to the field of moral psychology. The PD procedure was originally proposed by [18] to address the roles of recollection and familiarity-based guessing in memory performance. The central idea of the PD approach is to compare participants’ responses to incongruent and congruent moral scenarios. Deontological inclinations refer to inclinations to make moral judgments on moral obligatory, and they are closely associated with emotional reactions to harmful actions. Utilitarian inclinations refer to inclinations to make moral judgments on consequences of action, and they are related to trade-offs about costs and benefits [17].

Studies over the past decade have established that acute stress leads to more deontological moral judgment. However, the mechanism of how stress affects moral judgments is not fully understood. One possible way through which the effects of stress are accomplished might be emotional responses. Acute stress activates the sympathetic nervous system (SNS) and hypothalamic-pituitary-adrenal (HPA) axis, which in turn causes the release of hormones such as cortisol concentration and alpha-amylase, which inhibit our cognitive control-related brain areas and activate emotional processing-related brain areas. When participants are asked to make moral judgments in stressful situations, they will experience more negative emotions than in normal situations. There is a large body of research demonstrating that stress leads us to produce stronger intuitive emotional responses and the release of more endocrine hormones such as cortisol and α-amylase [19,20]. Emotional responses influence individuals’ moral judgments. According to the DPTMJ, the emotional response leads us to make more deontological judgments. Cushman et al. [21] reported that participants with higher aversive reactivity (indexed by peripheral vasoconstriction) tended to be not willing to endorse harmful actions. McDonald et al. [22] presented moral dilemmas to participants through 3D technology and asked them to make moral judgments. They found that participants with higher emotional arousal (skin electricity) were less likely to engage in harmful behavior and thus made more deontological moral judgments. Reynolds et al. [23] argued that individuals were reluctant to perform harmful actions because they anticipated negative emotions such as aversion associated with the action. Patil et al. [24] found that aversion to carrying out harmful actions partially mediated the relationship between psychopathy and utilitarian moral judgments. In addition, Li et al. [25] indicated that emotion regulation difficulties influenced moral judgments on five moral domains through the mediating role of emotional valence and arousal. Yet, few studies have directly examined the role of emotional responses in the association between acute stress and moral judgment. It is still unclear whether emotional responses mediate the relationship between stress and moral judgment.

To sum up, previous studies on the relationship between stress and moral judgments have mostly used the TSST paradigm to induce acute stress and employed a traditional data analysis approach for moral dilemmas. The standard TSST procedure always includes an arithmetic task, which has an impact on cognitive loads or resources, while manipulation of cognitive loads or resources alone could change the proportion of utilitarian judgments [26,27]. Therefore, The TSST paradigm may confound the effects of stress response and cognitive depletion on moral judgment. Moreover, the traditional moral dilemma analysis also has some shortcomings in separating and qualifying the relative strength of different moral inclinations. In addition, the role of emotional responses in the association between acute stress and moral judgment has not yet been examined directly. Therefore, the present study adopted the anticipatory stress paradigm to explore the influence of anticipatory stress on moral judgment and its underlying mechanism based on the PD approach. The anticipatory stress paradigm is adopted to induce stress with higher ecological validity. The PD paradigm is used to separate utilitarian and deontological inclinations to avoid confounding intrinsic motivation. Based on previous studies, we further explored the role of emotional arousal and valence in the relationship between anticipatory stress and moral judgment. Based on previous studies, we made the following hypotheses. (1) The anticipatory stress paradigm is effective in inducing acute stress, as reflected in both physiological and psychological indicators. (2) Anticipated stress leads individuals to make fewer utilitarian judgments. (3) Anticipated stress leads to an increase in deontological inclinations and no change in utilitarian inclinations. (4) Emotional responses mediate the relationship between stress and moral inclinations. Stress triggers emotional responses, and emotional responses further lead to changes in moral inclinations.

## 2. Methods

### 2.1. Participants

Participants were recruited on campus by means of advertisement, and some were excluded according to the exclusion criteria: neurological or psychiatric diseases, acute or chronic physical illness, extraordinary stressful life circumstances, or long-term drinking and smoking. Finally, 53 undergraduates attended our experiment. Among them, 20 were male and 33 were female, aged between 18–27 years (*M* = 21.66, *SD* = 2.03). These participants were randomly assigned to the stress group (N = 26) or the control group (*N* = 27). The two groups did not have significant differences in mean age (stress group *M* = 21.92, *SD* = 1.98; control group *M* = 21.41, *SD* = 2.08; *t* (51) = 0.92, *p* = 0.360, Cohen’s *d* = 0.257) and gender (*χ*^2^ = 0.011, *df* = 1, *p* = 0.915). To ensure accurate heart rate measurements, participants were asked not to perform strenuous exercise prior to participation in the experiment. All participants were required to sign an informed consent form before the start of the experiment, and they were debriefed and paid at the end of the experiment.

We did not calculate a sample size before collecting data. Instead, we followed the typical settings in previous publications. They usually included 20 to 25 participants in each group [5,11,12]. We performed post hoc power estimates using GPower 3.1 [28]. Given *η*^2^*p* = 0.174 (Cohen’s f = 0.459), α = 0.05, total sample size = 53, number of groups = 2, number of measurements = 2, corr among rep measurements = 0.09, nonsphericity correction ε = 1, we obtained a statistical power of 0.998 to probe a within-between interaction in a 2 (group) × 2 (parameter) repeated-measurement ANOVA. The power is much higher than the recommended criteria of 0.8. Therefore, the sample size is adequate in the present study for a repeated-measurement ANOVA.

### 2.2. Materials and Procedures

#### 2.2.1. Stress-Elicitation

The anticipatory stress elicitation paradigm we chose has been used in previous studies and its effects have been demonstrated [5,29]. It is more convenient to operate than the traditional Trier Social Stress Test (TSST). Following the anticipatory stress elicitation paradigm, participants in the stress group were informed to give a public speech in front of two experimenters on the topic of “How do I evaluate my cognitive abilities?” after the task of moral judgment. They would also be interviewed about the discrepancies between their self-reported performance and actual performance. They had three minutes to prepare their speech, but they were not allowed to take any notes. The presentation would be videotaped with a camera on the desk. When the preparation time (3 min) was up, they were asked to perform the moral judgment task. After that, they were told that they did not have to give a real speech. Participants in the control group were only told to recall the events of the previous vacation for three minutes.

#### 2.2.2. Measurements of Stress Response

The measurement of stress levels included both psychological and physiological indicators. The psychological indicators were obtained by means of a questionnaire, which was administered before and after the treatment for stress-elicitation, and the physiological indicators were obtained by electrocardiography (ECG) module of the BIOPAC MP150 throughout the whole experiment.

The State-Trait Anxiety Inventory (STAI). The State Anxiety subscale of the STAI was used to measure participants’ anxiety levels [30]. The subscale contains 20 items on a 4-point Likert-type scale from 1 (“not at all”) to 4 (“extremely”). The total score is obtained by summing the scores of the 20 items, ranging from 20 (lowest level of anxiety) to 80 (highest level of anxiety). This study used a Chinese version revised by Li et al. [31]. The Cronbach’s α coefficients in this study were 0.94 and 0.95 for the pre-treatment and post-treatment, respectively.

The Positive and Negative Affect Schedule (PANAS). The PANAS scale was used to measure participants’ levels of positive and negative affect [32]. The scale consists of two subscales, the Positive Affect subscale and the Negative Affect subscale, each with 10 items on a 5-point Likert-type scale from 1 (“not at all”) to 5 (“extremely”). The item scores of the two subscales were summed separately to indicate the current levels of positive and negative affect. The total scores for both subscales ranged from 10 (minimum) to 50 (maximum), with higher scores indicating more positive or negative affect. This study used a Chinese version of the PANAS scale revised by Wang et al. [33]. The Cronbach’s α coefficients of the PANAS were 0.76 and 0.71 for the pre-treatment and post-treatment, respectively.

Heart rate. Heart rate was recorded with the electrocardiography module (Electrocardiography, ECG) of the polyphysiometer BIOPAC MP150 system, and the data was analyzed using AcqKnowledge 5.0 software. ECG signal acquisition requires the use of an ECG100C amplifier, two LEAD110S shielded leads, one LEAD100 unshielded lead, and three disposable patch electrodes. Before testing, the three electrode patches were connected to the participant’s body, with the ground (GND) connected to the right lower limb, the positive (VIN+) connected to the left lower limb, and the negative (VIN-) connected to the left upper limb [34]. Participants were then asked to adjust to a comfortable position and to remain still during the experiment in order to accurately record the heart rate.

The Perceived Stress Scale (PSS-10). The PSS-10 scale was used to measure participants’ perceived chronic stress, which was severed as a control variable to ensure that chronic stress levels were the same between the stress group and the control group before acute stress was induced. The PSS-10 was developed from the 14-item version, and also indicated good psychometric properties [35]. The scale consists of 10 items. Participants were asked to indicate on a five-point Likert scale (from 0 (never) to 4 (very often)) about how often they felt or thought in the way each item described during the last month. The total score is obtained by summing the scores of the 10 items, ranging from 0 (minimum) to 40 (maximum), with a higher score indicating a higher level of chronic stress. This study used a Chinese Revision of the PSS-10 scale [36,37]. The reliability computed as Cronbach’s α coefficient was 0.87 in the current sample.

#### 2.2.3. Moral Dilemma Judgment and Emotional Response Measurement

Twelve moral dilemmas were adopted from two parallel forms of moral scenarios in B Gawronski, J Armstrong, P Conway, R Friesdorf and M Hütter [38]: (a) proscriptive norm that prohibits action and benefits of action greater than its costs; (b) proscriptive norm that prohibits action and benefits of action smaller than its cost. These two forms correspond to incongruent and congruent dilemmas in the PD analysis. A Chinese version of these moral dilemmas has been used in previous studies, e.g., [7,37]. We presented the moral scenarios one by one on the screen in a fixed random order. Participants were instructed to read each scenario, to rate their emotional valence (9-point Likert scale from 1 (very unpleasant) to 9 (very pleasant)) and emotional arousal (9-point Likert scale from 1 (very calm) to 9 (very excited)), and to rate the moral appropriateness of the agent’s utilitarian action (0 = inappropriate or 1 = appropriate.). The Cronbach’s α coefficient was 0.93 for the emotional valence rating, and 0.91 for the emotional arousal rating.

### 2.3. Procedure

**Figure 1:**
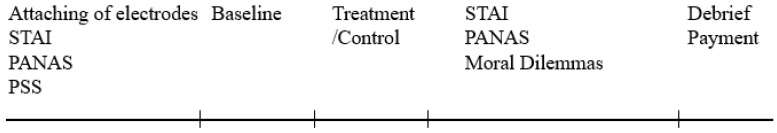
Procedures of the experiment. Note. STAI = State-Trait Anxiety Inventory; PANAS = Positive and Negative Affect Schedule; PSS = Perceived Stress Scale.

When participants arrived at the laboratory, they were first ensured that they did not engage in strenuous exercise, then they were asked to fill in informed consent forms, demographic information and questionnaires including STAI, PANAS, and PSS. After that, they were connected to the polysomnographic apparatus and were asked to sit still for 3 min and wait for the experiment to start. In the experiment, participants were randomly assigned to either the stress group or the control group, completed a 3 min stress elicitation procedure, and then completed the STAI and PANAS scales, followed by completing emotional response ratings as well as moral dilemma judgments, and finally, they were debriefed and paid (see Figure 1).

### 2.4. Statistical Analysis

To check whether anticipatory stress was successfully induced, we first performed a 2 (time) × 2 (group) repeated measures ANOVA on psychological indicators, i.e., scores of the STAI scale and PANAS scale, then conducted independent-sample *t*-tests to examine the differences on physiological indicators, i.e., heart rates. To check the effects of stress on moral judgments, we performed an independent sample *t*-test on the counts of utilitarian judgments under traditional analysis and a 2 (group) × 2 (parameter) repeated-measurement ANOVA under the process dissociation analysis. To examine the mediating roles of emotional valence and arousal, we conducted a series of mediation model analyses with the PROCESS macro for SPSS.

## 3. Results

### 3.1. Manipulation Check

Psychological indicators of stress. We conducted a 2 (time) × 2 (group) repeated measures ANOVA on scores of the STAI scale and PANAS scale, respectively, to test whether stress was induced as expected. Regarding anxiety levels, the time and group interaction was significant, *F* (1, 51) = 37.46, *p* < 0.001, *η*^2^*p* = 0.423. The Simple effects analysis showed no significant difference in anxiety levels between the stress and control groups before stress elicitation (stress group 34.88 ± 10.83; control group 35.81 ± 11.11; *F* (1, 51) = 0.095, *p* = 0.759, *η*^2^*p* = 0.002), but anxiety levels were significantly higher in the stress group than in the control group after the stress elicitation (stress group 52.38 ± 10.64; control group 35.48 ± 9.26; *F* (1, 51) = 38.15, *p* < 0.001, *η*^2^*p* = 0.428). Regarding positive affect, the time and group interaction was significant, *F*(1,51) = 11.72, *p* = 0.001, *η*^2^*p* = 0.187, and the results of the simple effects analysis indicated no significant difference in positive affect between the stress and control groups before stress was induced (stress group 31.42 ± 7.49; control group 30.78 ± 5.36; *F* (1, 51) = 0.131, *p* = 0.719, *η*^2^*p* = 0.003), but the level of positive affect was significantly lower in the stress group than in the control group after stress induction (stress group 27.04 ± 7.25; control group 32.04 ± 5.55; *F* (1, 51) = 7.98, *p* = 0.007, *η*^2^*p* = 0.135). Regarding negative affect, the time and group interaction was significant, *F* (1, 51) = 14.69, *p* < 0.001, *η*^2^*p* = 0.224, and the results of the simple effects analysis showed no significant difference in negative affect between the stress and control groups before stress was induced (stress group 14.92 ± 5.71; control group 15.15 ± 4.77; *F* (1, 51) = 0.024, *p* = 0.877, *η*^2^*p* = 0.000). However, after the stress induced, the level of negative affect was significantly higher in the stress group than in the control group (stress group 22.46 ± 7.78; control group 16.37 ± 4.23; *F* (1, 51) = 12.65, *p* = 0.001, *η*^2^*p* = 0.199) (see Table 1).

Physiological indicators of stress. Data on heart rates were calculated and analyzed using AcqKnowledge 5.0 software. First, we did an independent samples *t*-test for baseline heart rate in the stress and control groups, and the results indicated no significant difference in heart rates between the two groups prior to stress induction, *t* (51) = 1.151, *p* = 0.255, Cohen’s *d* = 0.322. We subtracted the baseline level from the heart rate during the stress induction phase, by this way to reduce individual differences. We then performed an independent samples *t*-test to compare the difference in stress levels between the stress group and the control group. The results showed that the changes in heart rate were significantly higher in the stress group than in the control group (stress group 11.687 ± 5.97; control group 0.752 ± 3.10; *t* (51) = 8.419, *p* < 0.001, Cohen’s *d* = 2.357).

Combining both psychological and physiological indicators of stress, the results indicate the success of stress manipulation.

### 3.2. Effects of Stress on Moral Judgments

Traditional analysis. The traditional analysis only includes incongruent moral dilemmas (or proscriptive norm that prohibits action and benefits of action greater than its costs). If participants indicated “appropriate”, a score of 1 was assigned, indicating a utilitarian judgment; if participants answered “inappropriate”, a score of 0 was assigned, indicating a deontological judgment. We calculated the sum of the “appropriate “scores for the six questions as the moral score. The results indicated a slight preference for utilitarian over deontological judgments (*M* = 3.528, *SD* = 1.54), and further results of the one-sample *t*-test indicated a significant difference between the overall preference and the median 3, *t* (51) = 2.50, *p* = 0.016, Cohen’s *d* = 0.343. An independent-sample *t*-test revealed that participants in the stress group (2.88 ± 1.75) would make less utilitarian judgments than participants in the control group (4.15 ± 0.99), *t* (51) = 3.25, *p* = 0.002, Cohen’s *d* = 0.893. The results supported the hypothesis that anticipated stress leads individuals to make more deontological judgments.

**Figure 2:**
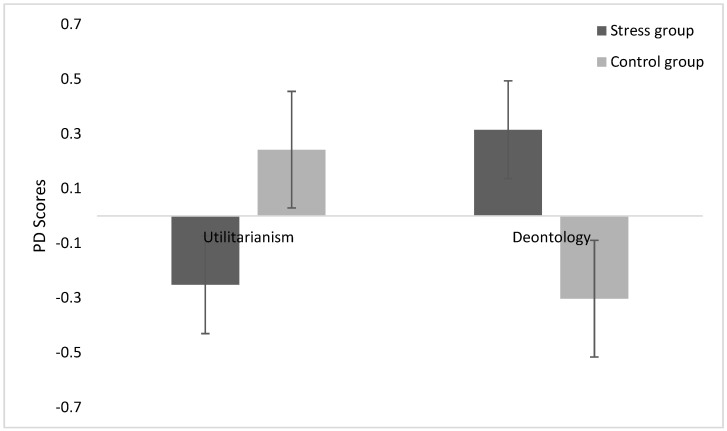
Mean standardized process-dissociation (PD) scores for the Stress and Control groups. Error bars represent standard errors of the mean.

Process dissociation (PD) analysis. The PD analysis includes both incongruent and congruent moral dilemmas. Following P Conway and B Gawronski [17], we calculated the raw utilitarian (U) parameter (utilitarian inclinations) and the raw deontological (D) parameter (deontological inclinations) and standardized the two parameters to obtain a common metric for mean comparison. We conducted a 2 (group) × 2 (standardized parameter) repeated-measurement ANOVA, which showed a significant interaction between group and standardized parameter, *F* (1, 51) = 10.74, *p* = 0.002, *η*^2^*p* = 0.174. Simple effect analysis showed that the stress group had significantly higher deontological inclinations (*M* = 0.32, *SD* = 1.09) than the control group (*M* = −0.30, *SD* = 0.82; *F* (1, 51) = 5.413, *p* = 0.023, *η*^2^*p* = 0.097). However, there was no significant difference between the stress and control groups in utilitarian inclinations (stress group *M* = −0.25, *SD* = 0.93; control group *M* = 0.24, *SD* = 1.03), *F* (1, 51) = 3.379, *p* = 0.072, *η*^2^*p* = 0.062) (see Figure 2). The results supported the hypothesis that anticipated stress leads to an increase in deontological inclinations and no change in utilitarian inclinations.

### 3.3. Mediating Role of Emotional Responses

Table 2 presents the descriptive statistics of major variables, including the mean, standard deviation, and correlations. We can see from the table that stress was significantly correlated with emotional valence (*r* = −0.798, *p* < 0.001), the standardized D parameter (*r* = 0.312, *p* = 0.023), and the utilitarian judgment (*r* = 0.446, *p* = 0.001), but not with emotional arousal (*r* = 0.041, *p* = 0.771) and the standardized U parameter (*r* = −0.249, *p* = 0.072). Emotional valence was significantly correlated with the standardized D parameter (*r* = −0.446, *p* = 0.001), but was not significantly correlated with the standardized U parameter (*r* = 0.103, *p* = 0.465). Both the D parameter and the U parameter were significantly correlated with utilitarian judgment, *r* = −0.585, *p* < 0.001, and *r* = 0.697, *p* < 0.001, respectively.

We examined whether stress could predict moral appropriateness rating, then we examined whether emotional valence mediated the association between stress and moral appropriateness rating. The bootstrapping Process for SPSS (Model 4, 5000 bootstrap samples) [39] was used to examine the mediation models.

For the standardized D parameter, the direct effect of stress on the D parameter was −0.238, 95% CI = [−1.072, 0.596], with the interval containing 0, and the indirect effect was 0.857, 95% CI = [0.196, 1.668], with the interval not containing 0. Therefore, emotional valence played a mediating role in the effect of stress on the D parameter.

For the standardized U parameter, the direct effect of stress on the U parameter was −0.915, 95% CI = [−1.807, −0.022], the interval did not contain 0, and the indirect effect was 0.421, 95% CI = [−0.396, 1.408], the interval contained 0. Therefore, the mediating effect of emotional valence between stresses on the U parameter was not significant (see Table 3).

The results supported the hypothesis that emotional valence mediates the relationship between stress and deontological inclinations. Stress triggers emotions with more negative emotional valence, and emotional responses further guide moral judgments.

## 4. Discussion

The present study explored the effect of anticipatory stress on moral judgment and the role of emotional responses in the association between acute stress and moral judgment. A manipulation check indicated that the anticipatory stress elicitation paradigm successfully induced acute stress both from psychological and physiological indicators. Moreover, stress has a significant effect on moral judgment. Specifically, the stress group made more deontological moral choices than the control group. The results of the PD analysis method indicated that stress increased deontological inclinations but it did not affect utilitarian inclinations. Mediation analysis further revealed that emotional valence mediates the association between stress and deontological inclinations.

The TSST paradigm was widely used in previous research exploring acute stress and moral judgment. This paradigm has been demonstrated to reliably induce acute stress. However, it might confound the effect of cognitive depletion (e.g., an arithmetic task) and the effect of stress [26]. In the present study, we employed an anticipatory stress paradigm to induce acute stress, following Starcke et al. [5]. Participants are told to give a speech after a moral judgment task, but they will not be required to perform a real speech after that task. This paradigm is effective in eliciting stress, as indicated by both physiological and psychological indicators. More relevant, our results are consistent with the findings of Starcke et al. [5]. They also demonstrated the effectiveness of the paradigm. However, Starcke et al. [5] suggested, the anticipatory stress paradigm involves anticipated speech rather than actual speech and arithmetic. This may induce a different type of response from that of the TSST paradigm, such as feelings of accountability.

Anticipatory stress leads to less utilitarian moral judgment. This finding is consistent with previous studies exploring the relationship between acute stress and moral judgment. Moreover, the finding is also consistent with that of Starcke et al. [5]. They also used the anticipatory stress elicitation paradigm and found that the stressed group made fewer utilitarian judgments than the control group. All those findings suggested that even anticipatory stress could lead to less utilitarian moral judgment. However, the traditional analytical method used in previous studies has some drawbacks, and leads to ambiguity in the interpretation of the results [17]. We further employed PD analysis techniques to address the problem. The results revealed that stress has an effect on deontological inclinations and no effect on utilitarian inclinations. It is consistent with findings in Li et al. [7], and supports our hypotheses. The findings are in line with the stress induced deliberation-to-intuition (SIDI) model proposed by Yu et al. [20] and the Dual Process Theory of Moral Judgment proposed by Greene et al. [16]. According to the SIDI model, individuals in stressful situations shift from a slow, rational analytic thought to a fast, intuitive emotional response, and thus participants make moral judgments based on negative emotional processing. The Dual Process Theory of Moral Judgment emphasizes that both cognition and emotion have important effects on moral judgment. The involvement of cognitive resources will make participants analyze rationally and focus on maximizing benefits, and thus make utilitarian judgments; while the involvement of emotional responses will make participants reluctant to commit harmful behaviors and follow rules, and thus make moral judgments, so that under stressful situations, participants will make more moral judgments.

Emotion plays an important role in many decision-making domains, including moral decision-making [25,40,41]. Based on previous research, we introduced emotional valence and emotional arousal as mediators to explore how stress affects moral judgment. We found that stress affects deontological inclinations rather than utilitarian inclinations through emotional valence. That is, stress induced more negative emotional valences and led to higher deontological inclinations. It did not affect utilitarian inclinations. This is consistent with the Dual-Process Theory of Moral Judgment [16,42]. Deontological and utilitarian inclinations correspond to two independent processes: a deliberative or cognitive process, and an automatic or emotional process. Their relative strength determines the final judgment [17]. However, our findings failed to identify a mediating role of emotional arousal. Therefore, the role of emotional arousal indicators needs to be further explored in the future.

Some limitations in the present study should be mentioned. First, we only focused on the roles of two aspects of emotion responses (i.e., emotional valence and emotional arousal), while other aspects or discrete emotions need to be considered in the future. Moreover, the process of stress affecting morality may also involve complex emotional-cognitive interactions. Second, neither traditional nor PD analysis for moral dilemmas can distinguish between deontological principles and general unwillingness to act tendencies or utilitarian principles and general willingness to act tendencies. The CNI model provides a potential solution to address this problem [38]. However, there are also some debates on the research paradigms, for example, the possible perverse responses in moral dilemma judgments [43,44,45]. Some researchers have attempted to address these limitations and further develop new paradigms [46,47,48]. Third, the moral dilemmas were related to life-and-death and presented in the form of text, which are still quite different from real-life moral dilemmas. Materials with higher ecological validity should be recommended, such as everyday moral dilemmas [3] and scenarios in virtual reality [49]. Finally, participants in the present study were students from a university in China. Generalization of the findings to other populations with different social-economic and cultural backgrounds should be done with caution [50]. Despite the above-mentioned limitations, the study has some valuable merits in theoretical and practical contributions to the field. We extended the exploration of the relationship between acute stress and moral judgment in two ways: (1) using anticipation rather than experiencing stressful events to induce anticipated stress; (2) introducing the PD paradigm to clarify interpretation ambiguities in traditional moral judgment analysis. We also further tested the role of emotional valence and emotional arousal in the relationship between stress and moral judgments, providing new insights into the effect of stress on moral judgment. Our study suggested that anticipatory stress led to increased negative emotional valence, increased deontological inclinations, and finally, resulted in fewer utilitarian moral judgments.

## 5. Conclusions

Anticipatory stress can be reliably induced with an anticipatory stress elicitation paradigm. It influences individuals’ performance in moral dilemmas. It triggers emotions with more negative emotional valence, then results in higher deontological inclinations, and finally leads to less utilitarian judgments.

## Figures and Tables

**Table 1:** Manipulation check for stress elicitation.

Stress Indicator	*F*	*df*	*p*	*η* ^2^ *p*
STAI: Time	34.71	1, 51	0.000	0.405
STAI: Group	10.34	1, 51	0.002	0.169
STAI: Time × Group	37.46	1, 51	0.000	0.423
PANAS-PA: Time	3.60	1, 51	0.064	0.066
PANAS-PA: Group	1.91	1, 51	0.173	0.036
PANAS-PA: Time × Group	11.72	1, 51	0.001	0.187
PANAS-NA: Time	28.25	1, 51	0.000	0.356
PANAS-NA: Group	4.71	1, 51	0.035	0.085
PANAS-NA: Time × Group	14.69	1, 51	0.000	0.224

Note. STAI = State-Trait Anxiety Inventory; PANAS = Positive and Negative Affect Schedule; PA = Positive Affect Schedule; NA = Negative Affect Schedule; Time = Pre-test and post-test; Group = the stress group and the control group.

**Table 2:** Stress, emotional response, and moral judgment using the PD approach.

Variable	M ± SD	1	2	3	4	5
Stress	0.49 ± 0.51					
Emotional valence	43.58 ± 15.04	−0.798 **				
Emotional arousal	59.04 ± 16.77	0.041	−0.137			
Standardized D parameter	0 ± 1	0.312 *	−0.446 **	0.096		
Standardized U parameter	0 ± 1	−0.249	0.103	0.173	0.094	
Utilitarian judgment	3.53 ± 1.54	−0.414 **	0.446 **	0.061	−0.585 **	0.697 **

Note. N = 53. Stress: 0 = control group, 1 = stress group. * *p* < 0.05, ** *p* < 0.01.

**Table 3:** Mediation analysis of emotional valence between stress and moral inclinations.

Parameters	Paths	Effect	SE	95% CI	
				LL	UL
D	Direct	−0.238	0.415	−1.072	0.596
	Indirect	0.857	0.377	0.196	1.668
U	Direct	−0.915	0.444	−1.807	−0.022
	Indirect	0.421	0.459	−0.396	1.408

Note. SE = standard error, CI = Confidence interval, LL = lower limit, and UL = upper limit.

## Data Availability

The raw data is publicly available at https://osf.io/zhu23/?view_only=6fa6463ad62e4729a6c87f07b90c5bf6 (accessed on 17 September 2022).
